# Measuring intersectional HIV, sexual diversity, and gender non-conformity stigma among healthcare workers in Ghana: scale validation and correlates of stigma

**DOI:** 10.1186/s12913-024-11098-6

**Published:** 2024-05-21

**Authors:** Emmanuel A. Oga, Melissa A. Stockton, Gamji R. Abu-Ba’are, Richard Vormawor, Emmanuel Mankattah, Stacy Endres-Dighe, Ryan Richmond, Sangchoon Jeon, Carmen H. Logie, Emma Baning, Khalida Saalim, Kwasi Torpey, Laron E. Nelson, Laura Nyblade

**Affiliations:** 1 RTI International, 3040 East Cornwallis Road, Research Triangle Park, NC 27709-2194 USA; 2 New York State Psychiatric Institute, Department of Psychiatry, Columbia University Irving Medical Center, 1051 Riverside Drive, New York, NY 10032 USA; 3 School of Nursing, University of Rochester Medical Center, University of Rochester, Rochester, NY USA; 4 Center for Interdisciplinary Research on AIDS, Yale School of Public Health, Yale University, New Haven, Connecticut, USA; 5 School of Nursing, Yale University, New Haven, CT 06520 USA; 6 Educational Assessment and Research Center, Accra, Ghana; 7 Factor-Inwentash Faculty of Social Work, University of Toronto, Toronto, ON M5S 1V4 Canada; 8 School of Public Health, University of Ghana, Accra, Ghana

**Keywords:** Intersectional stigma, HIV, MSM, Validation

## Abstract

**Background:**

Men who have sex with men (MSM) are at heightened risk for HIV acquisition, yet they may delay or avoid HIV testing due to intersectional stigma experienced at the healthcare facility (HCF). Few validated scales exist to measure intersectional stigma, particularly amongst HCF staff. We developed the *Healthcare Facility Staff Intersectional Stigma Scale (HCF-ISS)* and assessed factors associated with stigma in Ghana.

**Methods:**

We analyzed baseline data from HCF staff involved in a study testing a multi-level intervention to reduce intersectional stigma experienced by MSM. Data are from eight HCFs in Ghana (HCF Staff *n* = 200). The HCF-ISS assesses attitudes and beliefs towards same-sex relationships, people living with HIV (PLWH) and gender non-conformity. Exploratory factor analysis assessed HCF-ISS construct validity and Cronbach’s alphas assessed the reliability of the scale. Multivariable regression analyses assessed factors associated with intersectional stigma.

**Results:**

Factor analysis suggested an 18-item 3-factor scale including: *Comfort with Intersectional Identities in the Workplace *(6 items, Cronbach’s alpha = 0.71); *Beliefs about Gender and Sexuality Norms *(7 items, Cronbach’s alpha = 0.72); and *Beliefs about PLWH *(5 items, Cronbach’s alpha = 0.68). Having recent clients who engage in same-gender sex was associated with greater comfort with intersectional identities but more stigmatizing beliefs about PLWH. Greater religiosity was associated with stigmatizing beliefs. Infection control training was associated with less stigma towards PLWH and greater comfort with intersectional identities.

**Conclusions:**

Achieving the goal of ending AIDS by 2030 requires eliminating barriers that undermine access to HIV prevention and treatment for MSM, including HCF intersectional stigma. The HCF-ISS provides a measurement tool to support intersectional stigma-reduction interventions.

## Background

Biological, behavioral, legal, and socio-cultural factors place men who have sex with men (MSM) at high risk for contracting HIV [1–4]. Globally, MSM have an estimated risk of HIV infection 26 times that of the general population [5], where in sub-Saharan Africa, their risk of infection is 18 times higher [6], and in Ghana, from 8 to 11 times that of the general population [7, 8]. Among MSM, globally and in Ghana, stigma has been associated with increased fear or avoidance of health programs and reduced HIV testing.^1–4,9−11^ Stigma is a complex social process where personal attributes, whether real or perceived, are met by social exclusion, rejection, blame or devaluation [9]. For many, stigma serves as a significant barrier to accessing quality healthcare and consistently undermines progress towards ending major public health threats, including HIV. While there has been global progress in responding to HIV across the prevention to treatment care cascade [5], HIV stigma remains a significant barrier to ending the HIV epidemic and consistently shown to negatively affect HIV testing, care seeking and prevention and treatment engagement practices [10–13]. 

In addition, there is growing recognition within the HIV response of the need to understand, measure and respond to stigmas that intersect with HIV [14–16]. Intersectional stigma, a term first coined by Berger [17], has its roots in intersectionality theory and Black feminist scholarship and occurs when *“systems of power, privilege and oppression intersect to impact individual experiences and fuel stigma*” (pp. S356) [18] targeting multiple and interlocking social identities [19]. Different forms of social stigma (e.g., related to race, gender, sexuality, health status) intersect and compound, leading to a unique experience of discrimination that cannot be fully understood or addressed by considering each form of stigma in isolation. Intersectional stigma recognizes that individuals’ identities are multifaceted and that society can respond to these intersecting identities through imposing unique modes of discrimination and disadvantage that intensify the negative effects of stigma on health and wellbeing [20, 21]. As such, marginalized groups who are disproportionately affected by HIV, including MSM, face multiple and intersecting stigmas that compound their risk of HIV acquisition and further exacerbate barriers to HIV prevention and care [22, 23]. Ghanaian MSM face multiple and intersecting stigmas due to the association between MSM and HIV, cultural views towards same-gender attraction and sexual practices, perceived deviation from traditional gender roles and expressions for Ghanaian men, and an increasingly hostile political environment [24–28].

Understanding and responding to HIV and intersecting stigmas requires valid measures. In a recent review on measurement of intersectional stigma from the perspective of persons experiencing stigma, Karver et al. (2022) note that there is no consensus on how best to measure it and of the 16 studies that met study inclusion criteria, most explored intersectional stigma analytically via interaction terms between separately measured stigmas; only four did so through a single, intersectional measure. In addition, most studies were conducted in high income countries (primarily in the United States), capture one or two intersecting stigmas, and did not include any studies of community or organizational stigma. Measuring intersectional stigma towards marginalized populations from the perspective of persons within service provision organizations is also needed to estimate the prevalence of intersectional stigma within these organizations and collect data to inform and evaluate organizational focused interventions to reduce stigma.

While intersectional stigma can occur in all spheres of life, the presence of HIV-related intersectional stigma in health care facilities (HCF) is particularly damaging because these places are critical sources of HIV health information, counseling, testing, prevention and treatment services [29, 30]. Existing research in Ghana provides accounts of MSM being outright denied life-saving care due to their sexual identity, as well as gossiping, shaming, and outing, relating to intersecting identities of being MSM and living with HIV [24, 31]. In Ghana, preliminary work with HCF staff documented the presence of high levels of HIV and sexual stigma [32]. Overall, 57% of workers reported observing discrimination in their facilities towards people living with HIV (PLWH) in the past six months, while 40% reported observing discrimination towards MSM. HCF staff also perceived that their colleagues were unwilling to care for PLWH (47%) and MSM (44%). However, health facility stigma research and stigma-reduction interventions, both globally and in Ghana [14, 33], have largely focused on addressing only one type of stigma (e.g., only HIV stigma or only sexual stigma), without tackling the intersectional nature of the multiple stigmas encountered by marginalized clients, thereby overlooking the compound effects of intersectional stigma, especially relevant in diverse socio-cultural contexts like Ghana [34, 35]. This may in part be due to a lack of validated intersectional stigma measures for HCF staff.

In response, we describe the adaptation and validation of the *Healthcare Facility Staff Intersectional Stigma Scale (HCF-ISS)*, a scale to measure intersectional stigma (HIV, sexual and gender non-conformity) towards MSM among HCF staff in Ghana. Valid scales that measure intersectional stigma amongst HCF staff are essential to lay the groundwork for tailoring and evaluating interventions that take a more wholistic intersectional approach to reducing stigma amongst HCF staff to improve HIV prevention and care for MSM.

## Methods

### Study design and participants

This research is part of a parent study, the *Promoting Reductions in Intersectional StigMa (PRISM) in Ghana*, a mixed methods study that developed and evaluated a multi-level, intersectional stigma-reduction intervention in Ghana [36]. The intervention seeks to address the intersection of HIV, sexual, and gender non-conformity stigma faced by cisgender MSM at three socio-ecological levels—institutional (HCFs), interpersonal (within communities of MSM) and intrapersonal (individuals). The study was conducted in eight communities, four each, in the Greater Accra and Ashanti regions. The selected communities were clustered around the regional capitals as these had the highest proportion of MSM living with HIV in the country. A full methodological description of the primary trial is provided in the protocol paper [36] and descriptions of the multi-level interventions are published elsewhere [14, 22, 27]. MSM participants were eligible to participate if they were 18 years or older, assigned male sex at birth, self-identified as a cisgender man at time of enrollment and reported sexual activity with another man at least once within the previous six months. Transgender persons were not included. HCF staff employed in one of the 8 study facilities in a position with the opportunity to interact with MSM clients were included.

This paper focuses specifically on the adaptation and validation of a HCF intersectional stigma towards MSM measure utilizing only the baseline survey data collected in April 2021 from HCF staff in the 8 study facilities (*n* = 200, *n* = 25 per facility). The mixed methods parent study also included formative qualitative research to inform both intervention and measures adaptation, details of which have been previously published [14, 31]. 

HCF staff represented a diverse cross-section of the facility, who MSM are likely to encounter during the process of HIV testing (e.g., receptionist, security guard, nurse, HIV counselor, cashier). Purposive convenience sampling was deployed with 60% of HCWs recruited from departments MSM are more likely to visit (e.g., HIV clinic, outpatient, pharmacy, reception) and 40% from other departments. HCWs reporting to work first on data collection days in the selected departments were invited to participate, until the sample was reached. HCWs self-filled the paper questionnaire, with interviewers present to help if needed.

### Measures

*Stigma.* We were unable to identify an existing intersectional stigma scale focusing specifically on sexual diversity, HIV and gender non-conformity stigma towards cisgender MSM among HCF staff. Thus, guided by findings from the formative work [14, 22, 31], we adapted the Phobia and Attitude sub-scales of the LGBT Assessment Scale [37]. Specifically, we added: three parallel shame items for each focal stigma, namely sexual diversity, HIV and gender non-conformity; one HIV-specific blame item that captured personal responsibility for contracting HIV; and three items capturing preference to not provide services to three specific groups, namely MSM, PLWH and men with feminine mannerisms [38]. These items were also used to assess a HIV stigma-reduction intervention in Ghana [39]. It is important to note that in our paper, “men with feminine mannerisms” is a reference to cisgender men who do not conform to societal perceived norms of masculine gender expression. It does not refer to transgender individuals. We understand and accept that some individuals perceived to be men with feminine mannerisms are actually misgendered transgender women; however, many men with feminine mannerisms are cisgender. We also accept that some individuals who are perceived to be men, without feminine mannerisms, are misgendered transgender women. Our use of the term was specifically for men who are cisgender, but who did not adhere strictly to social expectations of masculinity. Our understanding that this is the most appropriate term for the phenomenon that we were trying to capture was based on both the formative research and the expertise of our community and health facility partners, including cisgender men with feminine mannerisms.

This adaptation exercise resulted in an initial set of 22 items that assessed opinions, beliefs, and attitudes towards MSM, PLWH and gender non-conforming cisgender men. Response options for all items fell on a 5-point Likert scale from strongly agree to strongly disagree; with 7 out of 22 items reverse-worded or scaled to reduce response bias. A higher score represents greater stigma. Specific adaptations to the Phobia and Attitudes sub-scales reflect the Ghanaian context, the intersectional stigma focus of the study (sexually diverse men, HIV and gender non-conformity) and the formative research findings. For example, we: replaced “gay man” with “men who have sex with men;” dropped the questions about bisexuality and replaced them with questions about PLWH; and replaced “transgender persons,” with “men with feminine mannerisms,” to capture cisgender MSM who do not conform to perceived norms of masculine gender expression in Ghana. We did not include an additional subset of questions about transgender individual both because the study was focused on cisgender MSM and formative findings indicated that the term transgender was not well understood by health facility staff and therefore would pose a challenge both for asking and interpreting the responses to the questions.

*Sociodemographic Data*: Additionally, HCF staff respondents provided data on their age, gender, educational achievement, the importance of religion, cadre (clinical versus non-clinical staff), number of years worked at the facility, type of clients served (e.g., clients living with HIV or clients who are MSM), and prior training (infection control, ethics, stigma, MSM-Friendly Services).

### Sample size considerations

The sample was a purposive sample for a total-facility stigma reduction intervention, and not primarily for the validation of the HCF-ISS. We conducted a factor analysis to validate a 22-item scale, with a sample size of 200 respondents. This sample size is considered adequate but on the lower threshold for factor analysis, especially given the larger number of items. While the rule-of-thumb of having 10 respondents per item was not fully met [40], previous research suggests that a minimum of 5 respondents per item can be sufficient for factor analysis under certain conditions, such as when the data quality is high, and the factor structure is strong and well-defined [41]. 

### Statistical analysis

All analyses conducted were cross-sectional. Descriptive analyses were conducted, and we calculated frequencies for sex, educational attainment, age category, job category and client history.

We conducted exploratory factor analysis (EFA) to assess construct validity and calculated Cronbach alpha to assess reliability of the HCF-ISS. We used EFA to identify the underlying structure of the original 22-item scale. It is particularly useful in this instance for assessing the construct validity of our scale and validate it for use in this population. We assessed factorability of the scale using the Kaiser-Meyer-Olkin measure of sampling adequacy, for which a score above 0.6 indicates whether a factor structure likely underlies the data. Bartlett’s test of sphericity for the correlation matrix was conducted to assess the existence of large correlations amongst the variables. A scree plot was created, in addition to observing eigen values, to identify the number of factors to be retained following initial factor analysis [42]. 

The details of exploratory factor analysis process are described in the Results section. Once number of factors were identified, we retained items with a significant critical factor loading (> 0.4), and which were not excessively cross-loaded across factors (> 0.3) We assessed convergent and divergent validity to understand the degree to which measures of constructs that theoretically should be related, based on EFA results, are in fact related [43]. Divergent validity on the other hand assessed whether concepts from EFA that are not supposed to be related are actually unrelated [44]. We conducted this analysis by evaluating the strength of the correlation coefficient between items in the factors and the composite score of their own factor, and that the correlations among items within the same factors are greater than correlations with items outside their own factors [45]. 

We conducted a multivariate regression analysis to assess the association between intersecting stigma targeting sexually diverse men, HIV and gender non-conformity stigma, and HCF staff characteristics. This provided insights on known-group validity which evaluates whether patterns of intersectional stigma reported on the HCF-ISS were in keeping with established knowledge. We used stepwise regression with forward selection for identification of covariates for the final regression models. Covariates were added to the model one at a time and all covariates with a p-value ≤ 0.2 were added to the final model. For the regression models sex, age, educational status, having prior infection control training, having MSM clients in the prior month, and religion importance were included as covariates.

## Results

The characteristics of the 200 respondents are provided in Table 1. Half of the participants were from the Greater Accra region and half were from the Ashanti region. Of note, 63% of participants were below the age of 35, 69% were female, and most (93%) had attained a tertiary level of education. In regard to cadre of staff, around three quarters were clinical staff and only a quarter were administrative or support staff. Only 39% had worked at their respective facility for more than five years. With respect to clients served in the last month, 17% had provided services to clients who were MSM, and 44% had provided services to 6 or more clients living with HIV. 52% of HCF staff indicated that religion was of “extreme importance” in their lives. In terms of prior training, 86% reported previous training in infection control, 88% reported previous training in ethics, 72% reported previous training on stigma, and only 24% reported previous training on MSM-Friendly Services.

**Table 1:** Participant Characteristics (*n* = 200)

Age	*n*	%
18–24	18	9.00
25–34	108	54.00
35–44	50	25.00
45–54	19	9.50
55+	5	2.50
**Sex**		
Male	62	31.00
Female	138	69.00
**Education**		
No formal education	1	0.51
Primary	4	2.02
Secondary/Technical/Vocational	9	4.55
Tertiary	184	92.93
**Religion of Extreme Importance** ^**#**^		
Yes	99	51.83
No	92	48.17
**Staff Category**		
Medical	146	74.11
Admin/Support	51	25.89
**Number of Years at Facility**		
> 5years	78	39.00
≤ 5 years	122	61.00
**Past-month MSM Clients**		
Had at least 1	32	16.93
None	157	83.07
**Past-month HIV Clients**		
1–5 Clients	109	56.48
6 or more clients	84	43.52
**Prior Training Received in**:		
Infection Control	169	85.79
Ethics	169	87.56
Stigma	140	72.16
MSM-Friendly Services	45	23.81

^#^Religion importance measured with question rated 1–9, 1 for not at all; and 9 for extremely important. The responses were categorized into “extremely important” vs. not

Exploratory Factor Analysis. Initially, the factorability of the 22 items on sexual diversity stigma, gender non-conformity stigma, and HIV stigma was examined. The Kaiser-Meyer-Olkin measure of sampling adequacy was 0.72, above the commonly recommended value of 0.6, and Bartlett’s test of sphericity was significant (χ2 (171) = 1157.903, *p* < 0.001).^49^

On factor analysis, initial eigen values indicated that the first three factors explained 44%, 23%, and 18% of the variance respectively. Solutions for two and three factors were each examined using varimax and oblimin rotations of the factor loading matrix. The three-factor solution, which explained 84% of the variance of the initial 22-item scale, was preferred because of: (a) its conceptual fit; (b) the ‘leveling off’ of eigen values on the scree plot after three factors; and (c) the insufficient number of primary loadings and difficulty of interpreting subsequent factors. The three factor varimax and oblimin solutions were both examined in subsequent analyses before deciding to use a varimax rotation for the final solution.

A total of four items were eliminated because they did not contribute to a simple factor structure and failed to meet a minimum criterion of having a primary factor loading of 0.4 or above, and no cross-loading of 0.3 or above. The 3-factor structure included: Factor (1) *Comfort with Intersectional Identities in the Workplace* with 6 items; Factor (2) *Beliefs about Gender and Sexuality Norms* with 7 items; and Factor (3) *Beliefs about People Living with HIV* with 5 items (Table 2). Two exceptional items were retained that did not meet the standard recommendations for factor loading and cross-loading. The item “*I would feel comfortable working closely with a person living with HIV*” cross loaded on both *Comfort with Intersectional Identities in the Workplace* and *Beliefs about People Living with HIV* (0.39 and 0.37 respectively), and not reaching the 0.4 threshold for factor loadings. This item was retained on *Comfort with Intersectional Identities in the Workplace* due to its strong conceptual fit with other items on that factor. Similarly, the item “*I would feel comfortable working closely with a man who has sex with men (MSM)*” did not reach 0.4 factor loading threshold but was retained for strong conceptual fit with other items on *Comfort with Intersectional Identities in the Workplace*. Additionally, the item “*Boys and men with feminine mannerisms are sinful*” was dropped from *People Living with HIV* for lack of conceptual fit with other items on that factor despite a strong factor loading of 0.58. In this instance, we favored conceptual fit because dropping the item did not adversely affect the overall factor structure negatively. The other 3 dropped items had excessive cross-loading. A factor analysis of the remaining 18 items was conducted with three factors explaining 91% of the variance of the *retained* scale. A varimax rotation provided the best-defined factor structure. The factor loading matrix for this final solution is presented in Table 2.

Internal consistency for each of the scales was examined using Cronbach’s alpha. The alphas were moderate: for *Comfort with Intersectional Identities in the Workplace* (0.71), for *Beliefs about Gender and Sexuality Norms* (0.72) and *Beliefs about People Living with HIV* (0.68). No substantial increases in alpha for any of the scales could have been achieved by eliminating more items.

For convergent validity: 61% of items (11 out of 18) have a correlation coefficient with the score of their own factor greater than 0.40; and for divergent validity: 100% of items have a correlation coefficient with the score of their own factor greater than those computed from the other factors. Composite scores were created for each of the three factors, based on the mean of the items which had their primary loadings on each factor. Items were reverse scored as appropriate and higher composite scores indicated greater levels of stigma.

Overall, these analyses indicated that three distinct factors were underlying HCF staff responses to the *HCF-ISS* items and that these factors were moderately internally consistent.

**Table 2:** Three-factor structure, factor loadings and correlations of the intersectional healthcare facilities stigma scale (*n* = 200)

Factors	Item No	Scale Items	Factor Loadings	Factor-item Correlations		
				Factor 1	Factor 2	Factor 3
**Comfort with Intersectional Identities in the Workplace** Cronbach’s Alpha = 0.71 Mean (SD) composite score = 2.1 (0.72)	1.	I would prefer not to provide services to people living with HIV	0.44	0.37	0.05	0.15
	2.	I would prefer not to provide services to men who have sex with men	0.81	0.56	0.13	0.15
	3.	I would prefer not to provide services to men with feminine mannerisms	0.89	0.66	0.06	0.15
	4.	I would feel comfortable working closely with a man who has sex with men (MSM)	0.30	0.37	0.16	0.19
	5.	I would feel comfortable working closely with a man with feminine mannerisms	0.50	0.38	0.03	0.30
	6.	I would feel comfortable working closely with a person living with HIV	0.39	0.47	0.17	0.13
**Beliefs about Gender and Sexuality Norms** Cronbach’s Alpha = 0.72 Mean (SD) composite score = 3.78 (0.65)	1.	If a person feels that they want to present their mannerisms, dress or practices in a different gender than the one they were born into (such as feminine presenting men), they should do everything to overcome these feelings	0.65	0.03	0.49	0.02
	2.	I would be ashamed if a boy or man in my family were MSM	0.57	0.19	0.48	0.12
	3.	I would feel that I had failed as a parent if I learned my son was MSM	0.54	0.03	0.48	0.10
	4.	Men who have sex with men threaten many of our basic social institutions	0.54	0.09	0.49	0.06
	5.	Men having sex with men is a sin	0.46	0.06	0.37	0.10
	6.	If a man has attraction/feelings for other men, they should do everything to overcome these feelings	0.51	0.07	0.36	0.12
	7.	Men with feminine mannerisms threaten many of our basic social institutions	0.44	0.28	0.35	0.30
**Beliefs about People Living with HIV** Cronbach’s Alpha = 0.68 Mean (SD) composite score = 2.3 (0.74)	1.	I would feel that I had failed as a parent if I learned that my child had HIV	0.60	0.09	0.09	0.51
	2.	People living with HIV threaten many of our basic social institutions	0.62	0.24	0.14	0.48
	3.	People living with HIV are sinful	0.47	0.14	0.03	0.37
	4.	I would feel ashamed if someone in my family was living with HIV	0.48	0.18	0.06	0.41
	5.	People living with HIV could have avoided HIV if they had wanted to	0.45	0.25	0.05	0.42

Mean stigma scores were computed for each factor by sex, age, education, job category and number of years spent working at the HCF as presented in Table 3. Overall mean (SD) composite scores for *Comfort with Intersectional Identities in the Workplace*, *Beliefs about Gender and Sexuality Norms*, and *Beliefs about People Living with HIV* were 2.1 (0.72), 3.8 (0.65) and 2.3 (0.74) respectively; with higher scores representing greater discomfort with intersectional identities in the workplace and stigmatizing beliefs about gender and sexuality norms, and about PLWH. We employed a straightforward mean scores approach for computing scores, focusing on simplicity. Also, Given the modest sample size and the need for computational feasibility, this method was deemed most appropriate. Although more nuanced methods like item response theory could potentially provide a deeper understanding of variations across domains, our approach ensures that our findings could be reliably replicated and applied in similar low-resource environments.

On univariable analysis, not having tertiary education was associated with greater discomfort with intersectional identities in the workplace: mean (SD) 2.6 (0.72) vs. 2.0 (0.70) for tertiary-level educated staff (*p* < 0.01). Being female was associated with greater stigmatizing beliefs about PLWH: mean (SD) 2.3 (0.75) vs. 2.1 (0.70) for males (*p* = 0.01); as was having less than 5 years of experience working at the facility: mean (SD) 2.3 (0.78) vs. 2.1 (0.66) for staff who have worked at the HCF for 5 years or longer.

**Table 3:** Mean stigma scores among HCF staff by gender, age, education, job category and number of years at facility (*n* = 200)

Mean (SD) Score Range: 0–4	Comfort with Intersectional Identities in the Workplace^#^	Beliefs about Gender and Sexuality Norms	Beliefs about People Living with HIV
**Sex**			
Male	2.2 (0.81)	3.8 (0.64)	2.1 (0.70)
Female	2.0 (0.67)	3.8 (0.67)	2.3 (0.75)
*p-value*	*0.33*	*0.86*	0.01*
**Age**			
18–34 years	2.1 (0.69)	3.8 (0.61)	2.3 (0.77)
35 years and over	2.0 (0.76)	3.8 (0.73)	2.2 (0.68)
*p-value*	*0.32*	*0.87*	*0.26*
**Education**			
Less than tertiary level	2.6 (0.72)	3.7 (0.75)	2.5 (0.58)
Tertiary level	2.0 (0.70)	3.8 (0.65)	2.3 (0.75)
*p-value*	< 0.01*	*0.62*	*0.31*
**Job Category**			
Administrative and support	2.2 (0.77)	3.7 (0.75)	2.3 (0.65)
Medical	2.0 (0.69)	3.8 (0.62)	2.2 (0.78)
*p-value*	*0.06*	*0.10*	*0.69*
**Number of Years at Facility**			
Less than 5 years	2.1 (0.68)	3.8 (0.66)	2.3 (0.78)
5 years or more	2.1 (0.77)	3.8 (0.65)	2.1 (0.66)
*p-value*	*0.75*	*0.89*	0.04*

^#^Higher scores represent greater *dis*comfort with intersectional identities in the workplace, stigmatizing beliefs about gender and sexuality norms, and about PLWH

Multivariate regression analysis was conducted including the following covariates: sex, age, educational status, having prior infection control training, having MSM clients in the prior month, and religion importance. As presented in Table 4, having prior infection control training, MSM clients in the past month, and a tertiary education were associated with less discomfort with intersectional identities in the workplace. Reporting greater importance of religion was associated with stigmatizing beliefs about gender and sexuality norms. Furthermore, being female was associated with stigmatizing beliefs about PLWH, while prior infection control training and having MSM clients in the past month were associated with more positive beliefs about PLWH.

**Table 4:** Multivariate analysis of association between stigma and HCF Staff characteristics

	**Comfort with Intersectional Identities in the Workplace**		**Beliefs about Gender and Sexuality Norms**		**Beliefs about People Living with HIV**	
	β (SE)	*p*-value	β(SE)	*p*-value	β(SE)	*p*-value
**Sex** (Reference: male)	-0.03 (0.12)	0.83	-0.04 (0.11)	0.74	0.35 (0.12)	< 0.01*
**Infection Control training** (Reference: no training)	-0.36 (0.15)	0.02*	-0.12 (0.14)	0.40	-0.46 (0.16)	< 0.01*
**Having MSM clients in past month** (Reference: none)	-0.40 (0.14)	< 0.01*	-0.16 (0.13)	0.23	0.32 (0.15)	0.04*
**Religion Importance** (Scored 0–9)	0.02 (0.03)	0.65	0.08 (0.03)	0.02*	-0.02 (0.03)	0.59
**Tertiary education** (Reference: no tertiary education)	-0.48 (0.24)	0.05*	-0.14 (0.22)	0.52	-0.11 (0.25)	0.67

- Higher scores represent greater *dis*comfort with intersectional identities in the workplace, stigmatizing beliefs about gender and sexuality norms, and about PLWH

## Discussion

Understanding and addressing intersectional stigma towards marginalized populations highly affected by HIV is recognized as key to ending AIDS by 2030 [46, 47]. Developing, targeting and evaluating intersectional stigma-reduction interventions requires valid measures. While the science of intersectional stigma measurement is progressing, there is no consensus yet about how best to measure it with most studies taking an analytic approach through interaction terms between separately measured stigmas, with a few developing single, intersectional measures [20]. In addition, most studies have been conducted in high income countries and from the perspective of those experiencing the stigma, rather than those enacting it—key targets for stigma-reduction interventions [48]. In this manuscript, we present the development and examination of the psychometric properties of the HCF-ISS, a novel instrument capturing HCF staff intersectional stigma (HIV, sexual and gender non-conforming) towards MSM. Our paper provides important additions to the literature given that it adds a new intersectional stigma measure for a marginalized population at higher risk of HIV (MSM), in a low-middle income country (Ghana) and focused on HCF staff, a critical group for stigma-reduction interventions given their critical role in HIV and other health services for MSM. Unlike existing tools for health care workers which have most often measured a single stigma [14, 38, 49–53], the HCF-ISS delves into the nuanced perceptions of sexual diversity, HIV, and gender non-conformity within the healthcare setting, providing a culturally contextualized understanding relevant to Ghana’s unique socio-political landscape. Additionally, the HCF-ISS will support stigma measurement to provide data to advocate for and facilitate policies and practices that are more inclusive and sensitive to the intersectional lives and needs of MSM.

The HCF-ISS has three distinct factors which demonstrated moderate convergent validity, strong divergent validity, and moderate internal consistency: Factor (1) *Comfort with Intersectional Identities in the Workplace*; Factor (2) *Beliefs about Gender and Sexuality Norms*; and Factor (3) *Beliefs about People Living with HIV*. The first factor comprised six questions about comfort in the workplace with colleagues who are MSM, PLWH, and men with feminine mannerisms. This provides evidence that stigma due to sexual identity, HIV, and gender-nonconformity are related and may be particularly relevant within the workplace. The second factor – *beliefs about gender and sexuality norms* – included both attitudes towards MSM and men with feminine mannerisms. This provides further evidence of the interlocking nature of stigma targeting sexual diversity and gender non-conformity. While often overlooked in stigma measurement, research has recognized that attitudes towards gender non-conformity may underlie and exacerbate sexual stigma [54–56]. However, at least one other study conducted in India that attempted to separately measure gender non-conformity stigma and sexuality stigma found evidence of multicollinearity and lack of conceptual clarity between the two constructs [55]. The third factor captures beliefs about PLWH, demonstrating that while HIV stigma persists alongside, and is related to, gender non-conformity and sexual diversity stigma, it has its own distinct aspects. This supports other conceptualizations of intersectional stigma and an ongoing scoping review is investigating this phenomena [22]. As a whole, our HCF-ISS is one of the first developed specifically for HCF staff.

In interpreting scores from the HCF-ISS, it is important to consider the potential variability in how stigma manifests across different identities. For example, a uniform score across different domains of stigma may suggest a generalized discomfort with marginalized identities among healthcare workers, necessitating broad-based educational interventions. Conversely, disproportionate scores may indicate specific areas of stigma that require targeted interventions. This nuanced approach to interpreting HCF-ISS scores underscores the scale’s utility in guiding intervention design, ensuring that efforts to reduce stigma are informed by a comprehensive understanding of its multifaceted nature.

Nyblade et al.’s HIV Stigma Framework posits that awareness, fear, attitudes, and the institutional environment drive manifestations of stigma in the HCF [46]. As such, we hypothesized that the factors in this intersectional stigma scale would yield similar associations. The multivariate analysis found that more stigmatizing beliefs about gender and sexuality norms (factor 2) were associated with placing greater importance on religion. This supports other findings that suggest religious beliefs contribute to stigmatizing attitudes, particularly in relation to MSM [57, 58]. Further, we found less stigmatizing beliefs about PLWH (factor 3) were associated with attending an infection control training. This supports theories that suggest experience and knowledge about the disease or identity, and in the case of HIV specifically that reducing fear of HIV acquisition in the workplace by building knowledge of infection control, will also shape stigmatizing attitudes [46]. Ultimately, the multivariate analysis also demonstrated that less discomfort with intersectional identities in the workplace (factor 1) was associated with having infection control training and tertiary education. Taken as a whole, these findings demonstrate the scale is performing in line with expectations set forth by HIV stigma frameworks and supports idea that known drivers – particularly awareness and stigmatizing attitudes – may drive discriminatory behaviors in HCFs [46, 59]. 

Increasingly, stigma-reduction research has found that greater contact with stigmatized groups can improve stigmatizing attitudes [33, 46, 60]. In this study, greater comfort with intersectional identities in the workplace (factor 1) and less stigmatizing beliefs about gender and sexuality norms (factor 2) were associated with providing services to clients who are MSM in the past month. As such, these findings suggest both factors are performing as expected and that increased contact with stigmatized groups can improve attitudes. Several recent literature reviews of stigma reduction interventions have noted that contact between stigmatized groups can improve attitudes and reduce stigma, particularly for HIV stigma [61–63]. However, in this study, we found that more stigmatizing beliefs about PLWH were associated with having MSM clients in the past month. This finding may initially appear surprisingly; however, it is possible that contact with MSM improves attitudes towards them as individuals but may reinforce preconceived notions and views towards HIV acquisition. Ultimately, while contact strategies may indeed offer an efficacious means of reducing stigma, these finding underly the futility of siloed approaches to stigma reduction and reiterates the importance of addressing all facets of the intersectional stigma faced by MSM.

Our study has some limitations. We utilized cross-sectional data, which limits the ability to draw causal inferences, however, cross-sectional data is useful for generating hypotheses for further exploration and can often be the only affordable and timely option in low resource settings. Additionally, because of a relatively small sample size, we were unable to perform confirmatory factor analysis as a follow up to exploratory factor analysis. Our study is conducted in urban and suburban facilities in Ghana, and the findings might not find applicability in rural settings, or in other settings outside of Ghana. Facility staff responses may have been subject to social desirability bias, which we do not account for in our analysis, however, stigmatizing beliefs and attitudes are evident in our findings.

Furthermore, we acknowledge limitations inherent to the additive approach used for the HCF-ISS. Specifically, this method may not fully capture the complex dynamics of intersectionality as highlighted by Turan et al. (2019), where different forms of stigma do not simply add up but interact in unique and unpredictable ways. Future iterations of the HCF-ISS will seek to incorporate measures that more directly assess these interactions, potentially through qualitative research methods or the development of items that specifically query about the intersection of stigmatized identities. Such improvements will aim to provide a richer, more nuanced understanding of intersectional stigma in healthcare settings.

## Conclusions

This study developed, validated and assessed the HCF-ISS, an 18-item scale that captures intersectional stigma towards MSM related to sexual diversity, HIV and gender non-conformity, among HCF staff in Ghana. The final scale captured three distinct factors: *Comfort with Intersectional Identities in the Workplace*; *Beliefs about Gender and Sexuality Norms*; and *Beliefs about PLWH*. Improving access to HIV prevention and treatment for MSM will require interventions that reduce intersectional stigma in HCFs, and specifically address these three key constructs. Given the increasingly hostile political climate in towards MSM in many countries in sub-Saharan Africa, including Ghana, fear of increased stigma and legal ramifications may further drive MSM underground, elevate their risk of contracting HIV, and exacerbate barriers to HIV services. Continued investment in understanding how intersectional stigma undermines HIV prevention and care as well as in how to eliminate it is increasingly critical and necessary. Along that vein, the HCF-ISS provides a valid and measurement tool to collect data essential to documenting the prevalence of intersectional stigma faced by MSM in HCFs as well as to advocate for, design and evaluate HCF stigma-reduction interventions. The findings of this study are particularly salient given the increasing socio-political hostility towards MSM in Ghana and other countries in sub–Saharan Africa. Our scale not only addresses a current gap in intersectional stigma measurement tools but also provides a timely tool for both researchers and practitioners in the region grappling with providing effective HIV services to MSM in the face of shifting cultural and legal dynamics.

## Data Availability

The datasets used and/or analyzed during the current study are available from the corresponding author on reasonable request.
